# *Staphylococcus caprae* Prosthetic Joint Infection Treated With Bacteriophage: A Case Report

**DOI:** 10.1155/cro/8034721

**Published:** 2026-09-09

**Authors:** Macy McPhee, Sara Strecker, Jennifer Hehl, Bethany Samperi, Dan Witmer

**Affiliations:** ^1^ Biology Department, University of Notre Dame, Notre Dame, Indiana, USA, nd.edu.au; ^2^ Department of Orthopedics, Bone and Joint Institute at Hartford Hospital, Hartford, Connecticut, USA; ^3^ Orthopedic Associates of Hartford, Hartford, Connecticut, USA

**Keywords:** bacteriophage, case report, recurrent periprosthetic joint infection, revision total knee arthroplasty, *Staphylococcus caprae*

## Abstract

**Background:**

Periprosthetic joint infection results in significant morbidity and mortality, often requiring multiple surgeries in conjunction with antibiotic therapy. While component exchange is often successful, in many cases the patient is too ill to undergo surgery. Bacteriophages are viruses that infect bacteria and are incredibly specific to their target, infecting bacterial cells without impact on the host. Success rates for treatment with bacteriophage approach 90%, but these treatments are not yet approved for widespread use.

**Case Presentation:**

We present a case of a 66‐year‐old male with a chronic prosthetic knee joint infection from *Staphylococcus caprae* treated with a combination of debridement, antibiotics, and implant retention (DAIR), long‐term antibiotics, and bacteriophage therapy, showing no recurrence and a significant reduction in pain at 2 years posttreatment.

**Conclusion:**

For recalcitrant prosthetic joint infections, treatment with bacteriophage as an adjuvant to care can drastically modify the clinical course.

## 1. Introduction

Periprosthetic joint infection (PJI) is a devastating complication of total joint arthroplasty, resulting in significant morbidity and mortality [1–5]. Management of PJI often includes multiple surgeries and prolonged antibiotic therapy, which can be very difficult for the patient [6]. In joint arthroplasty, bacterial biofilm can develop over the implanted prosthetic creating a complex microenvironment which, in conjunction with multidrug‐resistant microorganisms, leads to a high failure rate for procedures with only debridement and implant retention [7, 8]. Antibiotics alone, in conjunction with these procedures, are not able to penetrate the bacterial biofilm. Single‐ or two‐stage exchanges are reliable procedures for removal of biofilm wherein all implants are removed or exchanged; however, many clinical scenarios exist where these procedures are too morbid to perform safely.

Due to the high failure rate of antibiotics in these settings, alternative therapies such as bacteriophages have been proposed [7–10]. Bacteriophages are viruses that infect bacteria, resulting in the lysis of the target organism [8]. They are ubiquitous in nature and incredibly precise to their target, allowing for bacterial species‐specific targets [8, 9] that only infect the host bacterial cells without causing deleterious effects on the host microbiota [10]. As of September 2023, 246 patients have been treated with bacteriophages, of which 22 patients had a PJI [7]. Success rates approach 90% [9].

*Staphylococcus caprae* is a commensal, coagulase‐negative *Staphylococcus* species found in the skin flora of both humans and goats, from where the name originates [11, 12]. Most infections involving *S. caprae* occur in the lower limbs and are more likely to occur in men [11, 12]. *S. caprae* is also capable of creating a biofilm, allowing for the successful evasion of antibiotics and the chemotactic leukocyte response [11]. This report was prepared adhering to the CARE guidelines and focuses on a persistent PJI caused by *S. caprae*, which was treated with a bacteria species‐specific bacteriophage. At 2‐year follow‐up, the patient is doing well clinically with no recurrence of infection.

### 1.1. Statement of Informed Consent

The patient was informed that their data would be submitted for publication and provided written consent.

## 2. Case History

A 66‐year‐old male with a history of well‐controlled diabetes presented with a 7‐year history of a right knee PJI, having had a previous two‐stage exchange and multiple debridement, antibiotics, and implant retention (DAIR) revision procedures that had failed. A structured timeline for this patient can be seen in Table 1. Bilateral knee replacement was performed in January 2016. A geniculate nerve block was done in September 2016, and an ulcer was noted in August 2017. No aspiration was performed prior to revision surgery. The first stage of a two‐stage revision surgery for infection was performed in January 2018, 2 years after the initial surgery. Cultures at that time revealed *Staphylococcus not aureus*. The patient received IV Ancef for 6 weeks and was transitioned to daily minocycline (100 mg). The knee was reimplanted in May 2018, but the infection persisted, and the patient required another DAIR in June and September of that same year, due to a medial draining sinus. Cultures at that time grew *S. caprae*. He stopped antibiotics in June 2019. In February 2022, he presented with severe swelling, recurrent draining sinus tracts, and pain. He was switched from minocycline to cefedroxil (500 mg BID) in April 2022. He continued to have a draining sinus tract for the next 18 months, during which time several synovial fluid aspirates were attempted outpatient, but fluid was not able to be obtained. Additional interventions were not done during this 18‐month period as the patient was seeking second opinions from a variety of providers in the area. The patient was closely followed by the team every 4–6 weeks.

**Table 1:** Structured timeline showing previous procedures and bacteriophage treatment.

Timeframe/date	Event	Details and interventions
January 18, 2016	Bilateral TKA	Osteoarthritis diagnosis
		Recovered well but continued to wear a brace on the right knee
September 21, 2016	Nerve block	Genicular nerve block due to continuing pain in the right knee
		Radiofrequency lesioning in the right knee
August 20, 2017	Emergency visit	Cellulitis and ulcer noted on the right leg
		Treated by wound care until January 2018
January 28, 2018	1st stage of two‐stage revision for infection	Right knee only, antibiotic spacer placed
		Cultures: *Staphylococcus not aureus*
		IV Ancef for 6 weeks, then daily minocycline
May 30, 2018	2nd stage of two‐stage revision for infection	Reimplantation
		Placed on minocycline for 4 additional weeks
June 15, 2018	I&D	Minocycline continued post I&D
September 26, 2018	DAIR	Cultures: synovial fluid showed *Staphylococcus caprae*
		Intraoperative cultures negative
		Ancef treatment for prolonged period
		Minocycline therapy continued through 2019
February 3, 2022	Office visit for pain	No radiolucency or malalignment noted
		Joint swelling and sinus tract noted, minocycline therapy restarted
April 5, 2022	Severe swelling and draining sinus tracts	Minocycline discontinued; cefedroxil (500 mg BID) started
		Drainage stopped within 14 days of starting cefedroxil
October 20, 2022	Attempted aspiration	No fluid obtained
		X‐rays show well‐fixed implant
		Active drainage from sinus tract
November 2. 2022	Attempted aspiration with ultrasound guidance	No fluid obtained
		Active drainage from sinus tract
December 14, 2022	Attempted aspiration	No fluid obtained
		Active drainage from sinus tract
January 11, 2023	Follow‐up	No change in patient′s condition, followed every 4–6 weeks
June 30, 2023	Follow‐up	Open synovial biopsy
		Antibiotics stopped 2 weeks prior, then continued cefadroxil
		All cultures positive for *Staphylococcus caprae*
July–Sept 2023	Bacteriophage development/approvals	FDA compassionate use exemption granted
		Bacteriophage matched to patient sample
October 3, 2023	DAIR	All cultures positive for *Staphylococcus caprae*
		Bacteriophage administered via intra‐articular injection after washout (20 mL)
		IV vancomycin for 6 weeks postsurgery
October 4–8, 2023	Bacteriophage infusions (Cycle 1)	Daily bacteriophage IV infusions while in the hospital
October 18, 2023	Follow‐up	Wounds healed
		ESR and CRP still elevated; other labs normal
October 27, 2023	Bacteriophage IA injection	Bacteriophage administered via intra‐articular injection in office
October 27–31, 2023	Bacteriophage infusions (Cycle 2)	Daily bacteriophage IV infusions while outpatient
November 16, 2023	Follow‐up	No drainage, no pain
		IV vancomycin replaced with minocycline (100 mg BID) and cefadroxil (500 mg BID)
		CRP remains elevated but is decreasing
		ESR is now normal
December 4, 2024	Follow‐up	Right knee range of motion 10–90
		Well‐healed incision
		X‐rays show well‐fixed implant
		No pain, fevers, or chills
November 26, 2025	Follow‐up	Right knee range of motion 0–90
		Well‐healed incision
		Continues on cefadroxil (500 mg BID) and minocycline (100 mg BID)

As of June 2023, range of motion was 10°–90° actively and passively. His A1C was noted to be 6.7. The patient underwent an open arthrotomy synovial biopsy on his right knee in June 2023, during which several cultures were gathered. *S. caprae* infection was identified during this biopsy on every culture obtained using MALDI‐TOF mass spectrometry. Disc diffusion methods were used to confirm that the infection was susceptible to clindamycin, erythromycin, minocycline, tetracycline, and vancomycin. The patient′s infection was resistant to daptomycin, cefoxitin, and trimethoprim/sulfamethoxazole. Anaerobic and fungal cultures were negative at this time.

**Figure 1:**
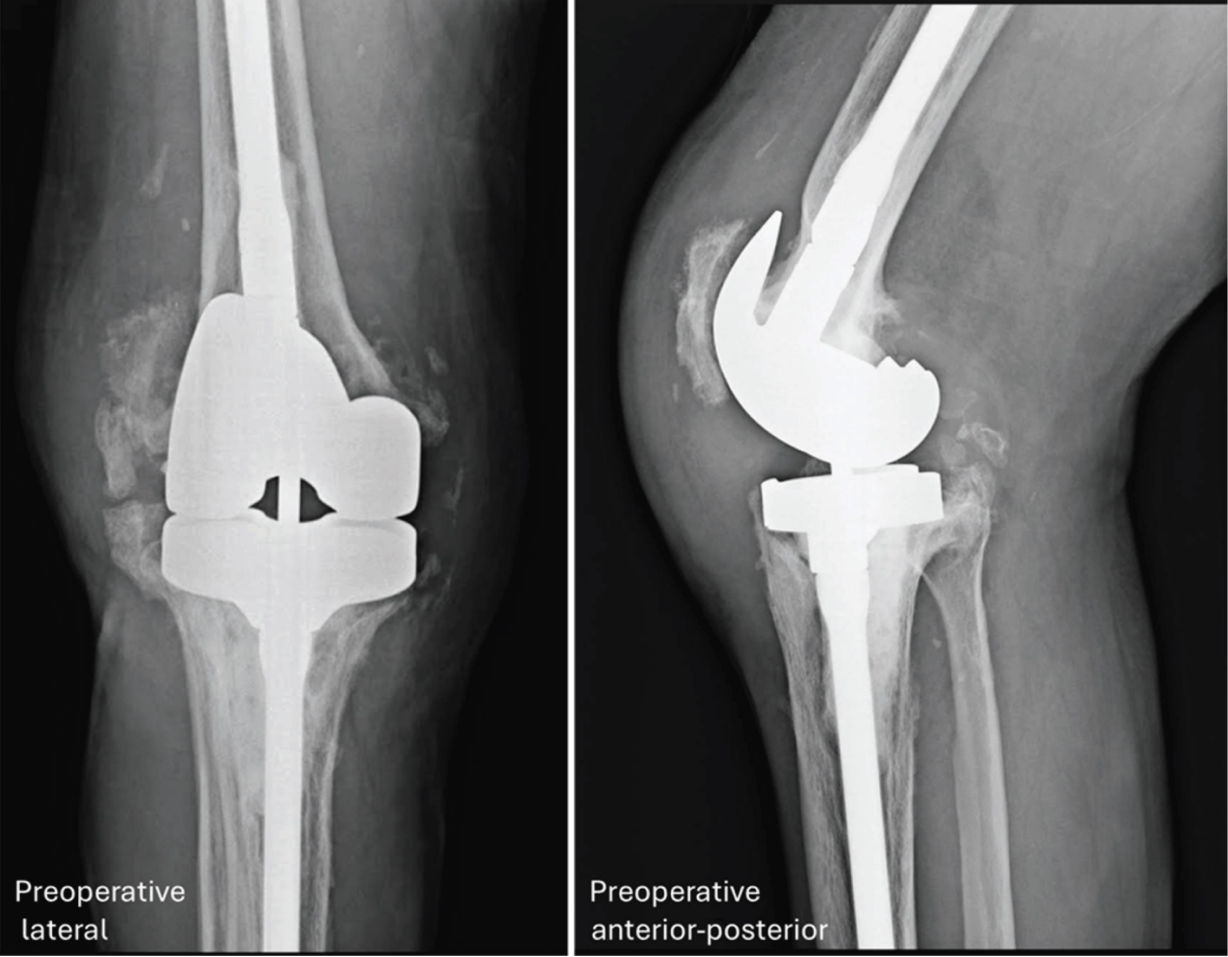
Preoperative x‐rays prior to the bacteriophage infusion showing well‐fixed stems but scalloping around the implant from persistent infection.

X‐rays taken prior to bacteriophage treatment demonstrated a well‐fixed revision TKA with stemmed implants and significant heterotopic bone noted (Figure 1). Small radiolucencies around the joint surfaces were unchanged when compared to x‐rays from 5 years prior with well‐fixated stems and no implant shift. A discussion was undertaken with the patient to review treatment options. Repeat DAIR was not recommended. The patient did not wish to try another two‐stage exchange, which also would have been quite morbid. A single‐stage exchange with extensive resection and possible megaprosthetic reconstruction was also declined, as were the options of fusion or amputation. Alternative therapies, including bacteriophage treatment, were discussed, which the patient was interested in pursuing through what was, at that time, an ongoing FDA trial at our center. A compassionate use exemption was granted from the FDA to utilize proprietary bacteriophage therapy on this patient, and patient samples were matched to an appropriate bacteriophage. This treatment strategy was proposed given the failure of prior two‐stage exchange and DAIR procedures, with the patient wishing to avoid fusion or above‐knee amputation.

Approximately 3 months following the open arthrotomy synovial biopsy, the patient underwent a DAIR procedure where bacteriophage was introduced directly into the infected knee. The bacteriophage used for this procedure was a single bacteriophage customized to this patient′s bacteria from a proprietary PhageBank that had been matched by genus, species, and morphology. The bacteriophage had a titer of 1–3 × 10^9^ PFU/mL (plaque‐forming unit) and was diluted in normal saline to achieve a final volume of 20 mL for the intra‐articular (IA) injection or 50 mL for postoperative IV administration. Additional information about the bacteriophage (bacteriophage matching, susceptibility testing, and bacteriophage characterization) is proprietary to the company and is therefore not available.

**Figure 2:**
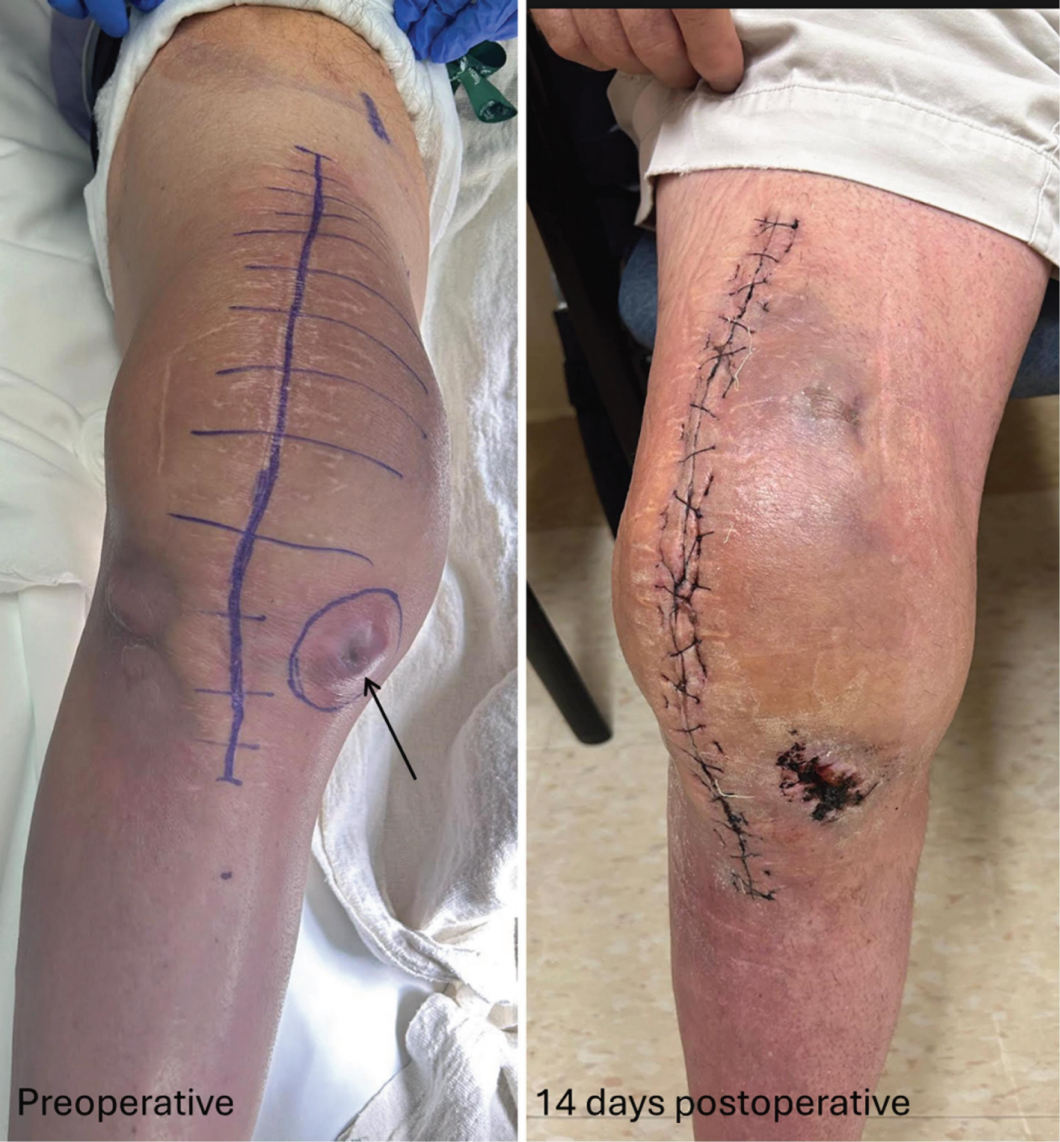
Preoperative and 14 days postoperative pictures of the right knee. The draining sinus tract is highlighted by a black arrow in the preoperative image. By 14 days postoperative, the sinus tract is closed, and wounds have healed.

Soft tissue status and prior incisions can be seen in Figure 2. Prior to the procedure, he had a C‐reactive protein (CRP) count of 4.70 mg/dL and an abnormal erythrocyte sedimentation rate (ESR) value of 42 mm/h. He had multiple abscesses around the knee and a medial draining sinus tract. During the procedure, all tissue appearing infected was removed, and additional samples were obtained intraoperatively for culture to compare with the screening culture results to confirm the identity of the bacteria. The patient had a pre‐existing Triathlon Total Stabilized (TS) Total Knee System. Femoral and tibial components were left in place as they were well fixed, and the modular polyethylene was removed and replaced (Figure 1). The TS poly component in the patient was replaced with one of a slightly smaller size to give him better motion given the tightness and scarring around the joint. The joint was then irrigated with 9 L of normal saline, followed by intraoperative bacteriophage administration, after which the incision was closed along with the excised sinus tract. Six weeks of IV antibiotics were started immediately after the procedure, and IV bacteriophage infusions were administered once daily for the next 4 days following the DAIR procedure while the patient remained in the hospital. This was considered the first cycle of treatment.

On Day 14, the wounds were noted to be completely healed (Figure 2), and the patient was given an additional single IA bacteriophage injection. This injection was followed by a daily outpatient IV bacteriophage infusion for the next 4 days. After 6 weeks, the patient was transferred from IV antibiotics to oral suppressive antibiotic therapy (SAT). This was considered the second cycle of treatment.

The patient was assessed regularly from the start of treatment through the end of the study, a duration of approximately 2 years. During this period, safety assessments were carried out, including evaluating infusion reactions, monitoring for adverse events (AEs), clinical laboratory testing, vital sign measurements, and physical examinations. Efficacy assessments were simultaneously implemented, including signs of infection, need for subsequent surgery, and recurrence of infection. Over the course of these assessments, the patient reported benefits from the treatment, noting decreases in pain and swelling, and there were no signs of recurrent infection. He denied experiencing adverse reactions to the IV infusions on each day upon receiving them. Four months after the beginning of treatment, the patient was stable, with minimal pain, able to walk unassisted, and the knee was not draining. No additional aspirates were taken after the bacteriophage treatment began. Patient reported outcomes were assessed initially and at 1 year. The patient′s KOOS, JR (Knee Injury and Osteoarthritis Outcome Score for Joint Replacement) improved from 42.3 to 68.3. The patient also reported that he experienced significant improvement on the Patient Global Impression of Change survey, going from very severe to very much improved between June 2023 and May 2024. Interestingly, he stopped his blood pressure (BP) medication as his chronically high BP normalized spontaneously around 3 months postsurgery. He had no dietary changes or weight loss during this time.

One year after the start of treatment, the patient′s improved status was maintained, with no cellulitis, only experiencing mild swelling and mild tenderness around the suprapatellar pouch. At this time, his CRP was 4.6 mg/dL, down from the value of 6.4 mg/dL as of 6 months prior, and his ESR was normal (2 mm/h). CRP and ESR levels from September 2018 through May 2024 can be seen in Table 2. Approaching 2 years since the beginning of treatment, he continued to take cefedroxil (500 mg BID) for his right knee as a precaution and experienced mild lateral joint discomfort with heavy activity. His knee incisions remained healed, and he felt significantly improved compared to his pretreatment state. He is considered Tier 2 according to the Musculoskeletal Infection Society (MSIS) workgroup criteria as he remains on suppressive antibiotics.

**Table 2:** Patient lab values prior to and during the year after his treatment.

Date	C‐reactive protein (mg/dL)	Erythrocyte sedimentation rate (mm/h)
9/25/2018	11.9	75
6/16/2023	4.7	38
9/27/2023	2.9	42
10/20/2023	8.8	33
11/21/2023	6.5	19
5/21/2023	4.6	2

## 3. Discussion

Chronic PJI is very difficult to treat, as a biofilm matrix often forms on the implants during the colonization of the bacteria [13–15]. Standard treatments include DAIR, single‐stage, and two‐stage exchange. These treatments can include multiple extensive surgeries, which put a significant burden on ill patients who commonly have significant medical comorbid issues [16]. After a failure of these standard treatments, some patients are left with an option of fusion or amputation. Bacteriophage therapy theoretically allows for the targeting of bacterial organisms in a very selective way, minimizing off‐target effects that can make other therapies, like systemic IV antibiotics, difficult to tolerate. Bacteriophages are believed to be able to penetrate and disrupt bacterial biofilm in areas that antibiotics alone cannot. The direct application of bacteriophage in conjunction with IV administration, as seen in this case report, has shown promise in several orthopedic applications [17–20], as most patients treated with bacteriophage have previously failed SAT and have undergone multiple surgical treatments, but while the treatment has been successful, bacteriophage treatment is still experimental and may not be directly causal. Due to the multiple concurrent interventions given to patients who have a PJI, it is very difficult to isolate the independent effect of the bacteriophage itself, and it is possible that the bacteriophage is working in conjunction with other treatment modalities to provide clinical remission.

Transition of bacteriophage therapy to routine practice is hindered by logical, regulatory, and scientific challenges, as the safety and efficacy of this treatment has not yet been adequately determined. Additionally, there are no publicly available PhageBanks in the United States which can be utilized by clinicians to appropriately match a patient′s bacteria to a bacteriophage, nor is manufacturing standardized [18, 19]. Clinical studies vary in treatment regimen, including dosing and delivery methods, which makes direct comparisons difficult. Even with these shortcomings, studies have shown that bacteriophages can interact synergistically with antibiotics and are generally considered to be safe, with minimal side effects like fever or erythema [19], with the existing literature supporting bacteriophage therapy as an adjuvant to SAT, especially in complex cases [20]. This case is similar to previously published PJI cases as all patients continued SAT post bacteriophage treatment, but unique in that a single bacteriophage was used, not a combination. This case is also the first report of a bacteriophage being used on a PJI caused by *S. caprae*. Bacteriophage therapy has been increasingly used in Europe [19, 21] with moderate success; however, additional safety and efficacy studies are needed to confirm results in a clinically rigorous way so that clinicians can appropriately utilize their potential and, if shown to be safe and effective, incorporate bacteriophage therapy into PJI treatment algorithms.

## 4. Summary

The use of a bacteriophage to treat a prosthetic joint infection is challenging, as bacteriophages are considered to be experimental and require an exemption from the FDA in order to use this treatment clinically in the United States. Additionally, all bacteriophages must be carefully matched to the organism in question, which in this case required an additional surgical procedure to obtain enough material to culture. Dosing and route of administration, and the bacteriophage identification number used, are not yet standardized in the literature. While this therapy appears safe, especially in cases where all other treatments have failed, larger studies are needed [22]. This report presents a case where recurrent PJI, treated with bacteriophage therapy and SAT, showed clinical remission for 2 years at this writing.

## Funding

No funding was received for this manuscript.

## Ethics Statement

The use of bacteriophage therapy was approved by the Hartford HealthCare Institutional Review Board (IRB# HHC‐2023‐0220, approved on 9/26/2023) and by special exemption from the FDA (IND# 29878, approved on 9/8/2023). The patient provided written informed consent for the use of bacteriophage therapy to treat his PJI and agreed to share his case in this report.

## Conflicts of Interest

The authors declare no conflicts of interest.

## Data Availability

The data that support the findings of this study are available on request from the corresponding author. The data are not publicly available due to privacy or ethical restrictions.
